# Postsynaptic Signals Mediating Induction of Long-Term Synaptic Depression in the Entorhinal Cortex

**DOI:** 10.1155/2008/840374

**Published:** 2008-07-22

**Authors:** Saïd Kourrich, Stephen D. Glasgow, Douglas A. Caruana, C. Andrew Chapman

**Affiliations:** ^1^Center for Studies in Behavioral Neurobiology, Department of Psychology, Concordia University, Montréal, QC, Canada H4B 1R6; ^2^Departments of Neuroscience and Psychology and the Institute of Human Genetics, University of Minnesota, Minneapolis, MN 55455, USA

## Abstract

The entorhinal cortex receives a large projection from the piriform cortex, and synaptic plasticity in this pathway may affect olfactory processing. In vitro whole cell recordings have been used here to investigate postsynaptic signalling mechanisms that mediate the induction of long-term synaptic depression (LTD) in layer II entorhinal cortex cells. To induce LTD, pairs of pulses, using a 30-millisecond interval, were delivered at 1 Hz for 15 minutes. Induction of LTD was blocked by the NMDA receptor antagonist APV and by the calcium chelator BAPTA, consistent with a requirement for calcium influx via NMDA receptors. Induction of LTD was blocked when the FK506 was included in the intracellular solution to block the phosphatase calcineurin. Okadaic acid, which blocks activation of protein phosphatases 1 and 2a, also prevented LTD. Activation of protein phosphatases following calcium influx therefore contributes to induction of LTD in layer II of the entorhinal cortex.

## 1. INTRODUCTION

The mechanisms that mediate the induction of long-term synaptic potentiation (LTP) [1, 2] and depression (LTD) [3–5] have been studied intensively within the hippocampus, but less is known about the signalling mechanisms for LTP and LTD in the entorhinal cortex. Because the entorhinal cortex receives highly processed inputs from sensory and association cortices and also provides the hippocampal region with much of its sensory input [6, 7], lasting changes in the strength of synaptic inputs to the entorhinal cortex could alter the manner in which multimodal cortical inputs are integrated, modulate the strength of transmission of specific patterns of sensory input within the hippocampal formation, and contribute to mnemonic function [8–11]. Determining the effective stimulation parameters and the intracellular signals that mediate synaptic plasticity in the entorhinal cortex should allow insight into basic mechanisms that contribute to the cognitive functions of the parahippocampal region.

Long-term potentiation of cortical inputs to the superficial layers of the entorhinal cortex has been described in vivo [11–14] and in vitro [15, 16]. Stimulation patterns required to induce LTP tend to be more intense in the entorhinal cortex than in the hippocampus [12, 14], and we have also found that induction of LTD in the entorhinal cortex requires intense low-frequency stimulation [17, 18]. In the hippocampus, conventional 1 Hz stimulation trains have been most effective in slices taken from juvenile animals [19, 20] but are generally ineffective in adult slices [21–23] and in intact animals ([31, 32], see also [33]). Similarly, 1 Hz stimulation induces entorhinal LTD in slices from young animals [28, 29] but is not effective in vivo [17] or in slices from older animals [18]. Repeated stimulation using pairs of pulses separated by a short 25- to 50-millisecond interval can induce LTD more effectively in both the CA1 ([24–26], but see [27]) and entorhinal cortex [17, 18, 33, 34]. In the CA1, the LTD induced by this stimulation pattern is NMDA receptor-dependent, but it also depends upon activation of local inhibitory mechanisms by the pulse-pairs [30, 31]. In the entorhinal cortex, however, repeated paired-pulse stimulation using a 10-millisecond interval that evokes maximal paired-pulse inhibition does not induce LTD, and LTD is induced when a 30-millisecond interval is used that evokes maximal paired-pulse *facilitation* [17]. The LTD can also be enhanced when GABA_A_ transmission is reduced with bicuculline [18]. This further suggests that LTD in the entorhinal cortex does not require activation of local inhibitory mechanisms but rather requires prolonged stimulation patterns that are strong enough to overcome local inhibition and lead to NMDA receptor activation. Strong local inhibition in the entorhinal cortex [8, 35] may thus place a restraint on activity-dependent synaptic modification. Consistent with this idea is the finding that the same pairing stimulation protocol that induces LTP in hippocampus leads to LTD in entorhinal cortex [28].

 Signalling mechanisms that mediate LTD in the superficial layers of the entorhinal cortex share some similarities with NMDA receptor-dependent LTD in the hippocampus. Long-term depression of superficial layer inputs to layer II is dependent on NMDA receptor activation both in vivo and in vitro [17, 18, 28, 33] but does not require activation of group I/II metabotropic glutamate receptors ([18, 28], see [36, 37]). In the hippocampus, moderate and prolonged influx of calcium via NMDA receptors activates calmodulin which leads to LTD via activation of the protein phosphatase calcineurin (PP2b). Calcineurin increases the activity of protein phosphatase 1 by reducing the activity of inhibitor 1, and this can cause rapid reductions in AMPA-mediated responses [2, 38, 39]. Hippocampal LTD is expressed partly through the reduced conductance of AMPA receptors caused by dephosphorylation of the GluR1 subunit by PP1 [2, 4], but careful study has shown that calcineurin-dependent LTD in deep layer inputs to layer II neurons in the young entorhinal cortex is not associated with a reduced AMPA conductance, but rather involves internalization of AMPA receptors and their proteosome-mediated degradation [28].

In the present study, the early postsynaptic signalling mechanisms that mediate LTD in layer I inputs to layer II neurons of the medial entorhinal cortex have been investigated using recordings of whole cell excitatory postsynaptic potentials. Long-term depression was induced using a prolonged paired-pulse stimulation pattern that was previously found to be effective for induction of NMDA-receptor-dependent LTD [18]. Pharmacological agents applied to the bathing medium or intracellular solution were used to assess the dependence of LTD on calcium-dependent signalling mechanisms including the phosphatases calcineurin and PP1/PP2a.

## 2. EXPERIMENTAL PROCEDURES

### 2.1. Slices and whole cell recordings

Experiments were performed on slices from male Long-Evans rats (4 to 8 weeks old). Animals were anesthetized with halothane and brains were rapidly removed and cooled (4°C) in oxygenated artificial cerebrospinal fluid (ACSF). ACSF consisted of (in mM) 124 NaCl, 5 KCl, 1.25 NaH_2_PO_4_, 2 MgSO_4_, 2 CaCl_2_, 26 NaHCO_3_, and 10 dextrose and was saturated with 95% O_2_–5% CO_2_. All chemicals were obtained from Sigma (St. Louis, Mo, USA) unless otherwise indicated. Horizontal slices (300 *μ*m) were cut with a vibratome (WPI, Vibroslice NVSL, Sarasota, Fla, USA) and were allowed to recover for at least one hour before recordings. Slices were maintained in a recording chamber with oxygenated ACSF at a rate of 2.0 mL/min, and a temperature from 22 to 24°C was used to minimize metabolic demands on slices [18, 28]. Neurons were viewed with an upright microscope (Leica DML-FS, Wetzlar, Germany) equipped with a 40x objective, differential interference contrast optics, and an infrared video camera (Cohu, 4990 series, San Diego, Calif, USA).

 Whole-cell current clamp recordings were obtained using patch pipettes pulled from borosilicate glass (1.0 mm OD, 4–7 MΩ) using a horizontal puller (Sutter Instr., P-97, Novato, Calif, USA) and filled with a solution containing (in mM) 140 K-gluconate, 5 NaCl, 2 MgCl_2_, 10 HEPES, 0.5 EGTA, 2 ATP-tris, 0.4 GTP-tris (pH adjusted to 7.25 with KOH). Tight seals (>1 GΩ) between the pipette and soma of cells in layer II of the medial entorhinal cortex were obtained in voltage-clamp, and whole-cell configuration was obtained using suction. Synaptic responses were evoked with a bipolar stimulating electrode constructed from two fine tungsten electrodes (1 MΩ; Frederick Haer & Co., Bowdoin, Me, USA) placed in layer I of the medial entorhinal cortex, 0.4 to 0.8 mm rostral to the recording electrode. Constant current pulses (0.1 millisecond, 60–250 *μ*A) were delivered using a pulse generator (WPI, Model A300) and stimulus isolator (WPI, A360). Responses to synaptic activation and intracellular current injection were obtained with an Axopatch 200B amplifier (Axon Instr., Sunnyvale, Calif, USA), filtered (10 kHz low-pass), displayed on a digital oscilloscope (Gould 1602), and digitized at 20 kHz (Axon Instr., Digidata 1322A) for storage on computer hard disc using the software package Clampex 8.2 (Axon Instr.). Series and input resistances were monitored regularly using −100 pA pulses. Recordings were accepted if the series resistance was <35 MΩ, and if input resistance and resting membrane potential were stable.

### 2.2. LTD Induction and pharmacology

Whole-cell current clamp recordings of EPSPs were monitored 10 minutes before and 30 minutes after LTD induction by delivering test-pulses every 20 seconds. Intensity was adjusted to evoke EPSPs that were approximately 3 to 4 mV in amplitude, and cells were held 5 mV below threshold when necessary to prevent the occurrence of spikes in response to EPSPs. Stimulus parameters for LTD induction were based on those used previously in vivo and in vitro [17, 18]. The induction of LTD was tested using pairs of stimulation pulses (30-millisecond interpulse interval) delivered at a frequency of 1 Hz for either 7.5 or 15 minutes [18]. Control cells received test-pulses throughout the recording period and did not receive conditioning stimulation.

 Signalling mechanisms mediating the induction of LTD were tested using stock solutions of pharmacological agents that were stored frozen and diluted on the day of use. NMDA glutamate receptors were blocked by constant bath application of 50 *μ*M DL-2-amino-5-phosphonovalerate (APV). The calcium chelator 1,2-bis(2-aminophenoxy)-ethane-N,N,N′N′-tetraacetic acid (BAPTA, 10 mM) was included in the recording electrode solution to block increases in intracellular calcium. To block activation of the calmodulin-dependent protein phosphatase calcineurin (PP2b) slices were pre-exposed to 250 *μ*M cyclosporin A (Toronto Research Chemicals Inc., North York, Ontario, Canada) for 1.5 to 3 hours [39]. In other experiments, FK506 (50 *μ*M) was included in the recording electrode solution to block calcineurin [39, 40]. In other experiments, okadaic acid (0.1 or 1.0 *μ*M) was included in the recording solution to block activation of protein phosphatases 1 and 2a [40, 41]. Control recordings without paired-pulse stimulation were used to verify the stability of recordings in cells filled with FK506 and 1.0 *μ*M okadaic acid.

### 2.3. Data analysis

Synaptic responses and electrophysiological properties of layer II neurons were analyzed using the program Clampfit 8.2 (Axon Instr.). Data were standardized to the mean of baseline responses for plotting and were expressed as the mean ±SEM. Changes in EPSP amplitude were assessed using mixed-design ANOVAs and Neuman-Keuls tests that compared the average responses during the baseline period, 5 minutes after conditioning stimulation, and during the last 5 minutes of the recording period.

Layer II neurons were classified as putative stellate or nonstellate neurons based on electrophysiological characteristics described by Alonso and Klink [42]. Stellate neurons were characterized by the presence of low-frequency subthreshold membrane potential oscillations, a depolarizing afterpotential following spikes, and prominent inward rectification in response to hyperpolarizing current pulses. Both pyramidal and stellate neurons in layer II can show inward rectifying sag responses [43]. Here, neurons recorded were clearly in layer II, usually near the border with layer I, and a proportion of these neurons did not show clear sag and were classified as pyramidal neurons. Input resistance was determined from the peak voltage response to −100 pA current pulses (500-millisecond duration), and rectification ratio was quantified by expressing peak input resistance as a proportion of the steady-state resistance at the end of the current pulse.

## 3. RESULTS

Stable recordings were obtained from 57 putative stellate neurons and 21 putative nonstellate cells. Peak input resistance was similar in stellate and pyramidal neurons (stellate, 95 ± 6 MΩ; pyramidal, 96 ± 10 MΩ) but there was a much larger sag in voltage responses to hyperpolarizing current injection in stellate cells (rectification ratio 1.37 ± 0.04 in stellate cells versus 1.06 ± 0.01 in pyramidal cells). The amplitude of baseline synaptic responses evoked by layer I stimulation was similar in stellate (3.9 ± 0.2 mV) and pyramidal cells (3.7⁢ ± 0.4 mV), and the amount of depression induced was also similar for recording conditions in which significant LTD was obtained (71.2 ± 5.6% in 14 stellate and 76.8 ± 7.6% in 6 pyramidal cells).

### 3.1. LTD induction

**Figure 1:**
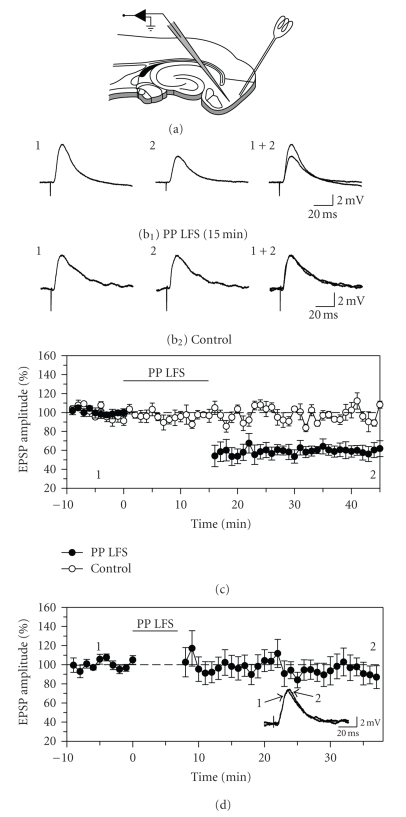
Prolonged, low-frequency stimulation induces long-term depression of EPSPs in neurons in layer II of the entorhinal cortex. (a) The location of stimulating and recording electrodes in acute slices containing the entorhinal cortex. (b) and (c) Long-term depression was induced by repetitive delivery of pairs of stimulation pulses at a rate of 1 Hz for 15 minutes (PP-LFS). The amplitude of synaptic responses remained stable in control cells that did not receive conditioning stimulation. Traces in (b) compare responses recorded during the baseline period (1) and during the follow-up period (2) in a neuron that received low-frequency stimulation (b_1_) and in a control cell (b_2_). Responses were obtained at the times indicated in (c). Averaged points in (b) indicate the mean ±1 SEM in this and subsequent figures. (d) Long-term depression was not reliably induced when low-frequency stimulation was delivered for only 7.5 minutes rather than 15 minutes, indicating that induction of LTD requires prolonged stimulation.

To determine if a relatively brief LTD induction protocol could be used to induce LTD in whole-cell recordings, the first tests attempted to induce LTD using paired-pulse delivery at 1 Hz for 7.5 minutes (*n* = 10) which can induce moderate LTD of field potentials in a gas-fluid interface recording chamber [18]. Paired-pulse stimulation for 7.5 minutes did not induce depression of EPSPs relative to control cells (93.0 ± 10.0% of baseline after 30 minutes; F_2,28_ = 0.09, *P* = .92). We previously observed stronger LTD of field potentials in the interface recording chamber after 15 minutes versus 7.5 minutes of paired-pulse stimulation [18], and prolonged paired-pulse stimulation for 15 minutes also reliably induced LTD of whole-cell EPSPs (*n* = 7, Figure 1). EPSP amplitude was reduced to 56.3 ± 9.5% of baseline levels 5 minutes after the conditioning stimulation, and remained at 58.6 ± 6.1% of baseline levels at the end of the 30 minutes follow-up period (F_2,22_ = 14.2, *P* < .001). Responses in control cells were stable (*n* = 6), and remained at 99.6 ± 2.6% of baseline levels at the end of the recording period (Figures 1(b_2_), 1(c)).

### 3.2. NMDA receptors and postsynaptic calcium

**Figure 2:**
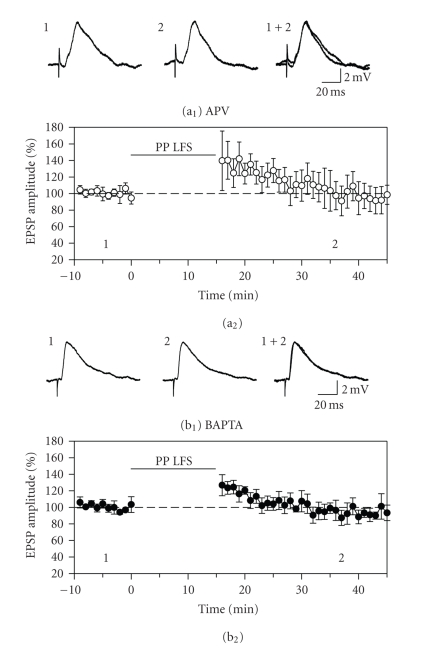
The induction of long-term depression is dependent on activation of NMDA glutamate receptors and on increases in postsynaptic calcium. (a) Constant bath application of the NMDA receptor antagonist APV (50 *μ*M) blocked the induction of long-term depression by 15 minutes of paired-pulse low-frequency stimulation (PP LFS). (b) Blocking increases in postsynaptic calcium by including the calcium chelator BAPTA (10 mM) in the recording electrode solution also blocked the induction of LTD. The transient facilitation of EPSPs immediately following stimulation was significant for the BAPTA condition but not the APV condition, and responses were at baseline levels at the end of the recording periods. The block of lasting depression suggests that calcium influx via NMDA receptors is required for induction of LTD.

The NMDA receptor antagonist MK-801 blocks induction of LTD in the entorhinal cortex in vivo [17] and the NMDA receptor blocker APV has been shown to prevent LTD of field potentials and EPSPs in entorhinal cortex slices [18, 28, 33]. We therefore tested for the NMDA receptor-dependence of LTD of EPSPs in the current preparation using constant bath application of APV (50 *μ*M). Induction of LTD by 15 minutes of paired-pulse stimulation was blocked by APV (*n* = 6, Figure 2(a)). There was a tendency for responses to be potentiated immediately following conditioning stimulation, but this variable effect was not statistically significant, and responses were close to baseline levels at the end of the recording period (96.7 ± 13.2% of baseline; F_2,10_ = 2.99, *P* = .09).

The role of postsynaptic calcium in LTD induction was tested by recording from cells in which the calcium chelator BAPTA (10 mM) was included in the recording electrode solution (10 mM, *n* = 6, Figure 2(b)). Cells filled with BAPTA had longer-duration action potentials than control cells (6.1 ± 0.7 versus 3.3 ± 0.1 milliseconds measured at the base; *t*_1,9_ = 3, 57, *P* < .01) consistent with a reduction in calcium-dependent potassium conductances. The induction of LTD was blocked in cells loaded with BAPTA. There was a significant increase in the amplitude of EPSPs immediately following paired-pulse stimulation (to 122.3 ± 6.0% of baseline; F_2,10_ = 5.46, *P* < .05; N–K, *P* < .05), but responses returned to baseline levels within 10 minutes and were at 94.8 ± 7.1% of baseline levels after 30 minutes (N–K, *P* = 0.50, Figure 2(b)). An increase in postsynaptic calcium is therefore required for induction of LTD in layer II neurons of the entorhinal cortex.

### 3.3. Protein phosphatases

**Figure 3:**
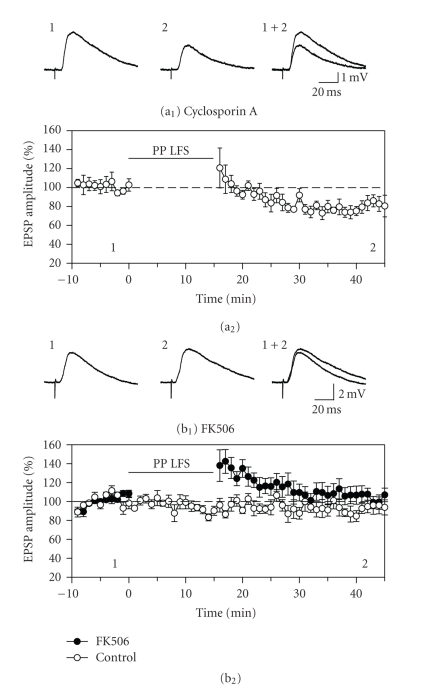
Long-term depression is dependent on activation of the calmodulin-dependent protein phosphatase calcineurin. Although LTD was only partially inhibited by pre-exposure to cyclosporin A, it was completely blocked when FK506 was included in the recording electrode solution. (a) Pre-exposure of slices to the calcineurin inhibitor cyclosporin A (250 *μ*M) for 1.5 to 3 hours resulted in a partial block of LTD by repeated paired-pulse stimulation. The amount of LTD induced was smaller than in control ACSF and was close to statistical significance (*n* = 6, *P* = .07). (b) Including the FK506 in the recording electrode solution to directly block postsynaptic calcineurin prevented the induction of LTD. Analysis of group responses showed a significant increase in responses during the baseline period, but responses in control cells indicate that this increase is transient and unlikely to have affected measurement of LTD. Inhibition of postsynaptic calcineurin therefore prevents induction of LTD in layer II cells of the entorhinal cortex.

The role of the calmodulin-dependent protein phosphatase calcineurin (PP2b) in LTD in layer II neurons was tested using either pre-exposure to 250 *μ*M cyclosporin A in the bathing medium [39], or by including 50 *μ*M FK506 postsynaptically in the recording electrode solution. In cells pre-exposed to cyclosporin A, paired-pulse stimulation was followed by a depression in EPSP amplitude that reached 82.4 ± 7.5% of baseline levels after 30 minutes (Figure 3(a)). Although the depression in the cyclosporin group was not statistically significant (F_2,10_ = 3.51, *P* = 0.07, *n* = 6), the depression obtained was also not significantly less than that observed in control ACSF (F_1,11_ = 3.79, *P* = .08). The result was therefore ambiguous with respect to the role of calcineurin in LTD. To test the involvement of calcineurin more definitively and to avoid potential presynaptic effects, the calcineurin blocker FK506 was included in the recording electrode solution for additional groups of cells [40]. Responses in cells filled with FK506 showed a significant potentiation immediately following paired-pulse stimulation (*n* = 8), but there was no lasting change in response amplitudes in comparison to control cells filled with FK506 that did not receive conditioning stimulation (*n* = 7). Responses were increased to 134.9 ± 10.5% of baseline levels immediately following paired-pulse stimulation, (F_2,26_ = 7.71, *P* < .01; N–K, *P* < .001; *n* = 8) but returned to 102.2 ± 6.1% of baseline levels after 30 minutes (Figure 3(b)).

Inspection of averaged responses suggested that there was an initial increase in responses during the baseline period among cells filled with FK506, and comparison of responses recorded during the first and last minutes of the baseline period showed that the increase was significant (*t*_14_ = 3.09, *P* < .01). Interestingly, then, interfering with calcineurin function can lead to enhanced basal synaptic transmission in entorhinal neurons. This increase is not likely to have affected measures of LTD in conditioned cells, however, because control responses showed only a transient increase after which responses remained stable.

**Figure 4:**
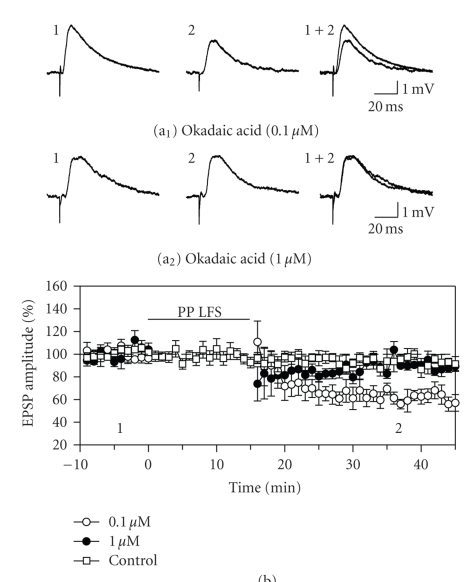
The induction of LTD was blocked in a dose-dependent manner by including okadaic acid in the recording electrode solution to block activation of protein phosphatase 1 (PP1). (a) and (b) A low concentration of 0.1 *μ*M okadaic acid failed to block LTD induction, but raising the concentration to 1.0 *μ*M resulted in a block of LTD induction (compare traces in A_1_ versus A_2_). Responses in control cells filled with 1.0 *μ*M okadaic acid that did not receive conditioning stimulation remained stable. The block of LTD by okadaic acid suggests that activation of PP1 mediates LTD in the entorhinal cortex.

Protein phosphatase 1 is thought to contribute directly to suppression of hippocampal EPSPs during LTD by dephosphorylation of the GluR1 AMPA receptor subunit. The involvement of PP1 to LTD in the entorhinal cortex was therefore tested by including okadaic acid in the recording electrode solution. In early experiments, a low concentration of 0.1 *μ*M okadaic acid [41] did not block LTD induction, and responses were depressed to 72.7 ± 8.7% of baseline levels at the end of the recording period (F_2,24_ = 4.65, *P* < .05; N–K, *P* < .001; *n* = 8). However, increasing the concentration of okadaic acid to 1.0 *μ*M [40] blocked the induction of LTD. There was a variable and nonsignificant reduction in responses immediately following conditioning stimulation (to 89.0 ± 14.9% of baseline) and responses were also near baseline levels after 30 minutes (96.0 ± 6.6% of baseline 30; F_2,22_ = 0.18, *P* = .84; *n* = 7; Figure 4). Activation of PP1 is therefore likely to contribute to mechanisms of LTD in the entorhinal cortex.

## 4. DISCUSSION

The current paper has used prolonged repetitive paired-pulse stimulation to induce LTD in layer I inputs to layer II neurons of the medial entorhinal cortex and has determined the early postsynaptic signals that mediate LTD in these cells. Consistent with previous observations, the LTD observed here was obtained in both putatively identified stellate [28] and pyramidal [44] cells. The induction of LTD was blocked by the NMDA glutamate receptor antagonist APV, and by the calcium chelator BAPTA, indicating that calcium influx via NMDA receptors is required for LTD. The induction of LTD was also blocked by the calcineurin inhibitor FK506, and by okadaic acid which blocks activation of protein phosphatases 1 and 2a. Calcineurin is required for LTD of deep layer inputs to layer II stellate cells [28], and calcineurin-dependent activation of PP1 contributes to NMDA receptor-dependent LTD of AMPA responses in the hippocampus [2, 4].

The dependence of LTD in the entorhinal cortex on activation of NMDA receptors has been a consistent finding in vivo and in slices. It has been observed following stimulation protocols including 1 Hz trains, pairing of presynaptic stimulation at 0.33 Hz with postsynaptic depolarization [28], repeated paired-pulse stimulation [18, 33], and spike-timing-dependent induction of LTD [44]. Long-term depression was blocked by including the calcium chelator BAPTA in the recording electrode solution (Figure 2) [28], and this is consistent with calcium influx via NMDA receptors as a critical trigger for entorhinal LTD. Metabotropic glutamate receptor activation and release of calcium from intracellular stores can contribute to LTD in the hippocampus [2, 36, 37, 45], but activation of metabotropic glutamate receptors is not required for entorhinal LTD [18, 28]. Calcium influx through voltage-gated calcium channels can contribute to spike-timing-dependent LTD in the entorhinal cortex, however. Cells with broadened action potentials that result in larger calcium transients show greater NMDA receptor-dependent spike-timing-dependent LTD in layer II-III cells [44]. Calcium influx through voltage-gated channels also mediates bidirectional spike-timing-dependent plasticity of inhibitory synapses in entorhinal cortex [46]. A form of long-term depression on layer V-VI neurons, expressed presynaptically through reduced transmitter release, is also dependent on activation of voltage-dependent calcium channels [33]. Calcium signalling mediated by voltage-gated channels therefore plays a number of roles in modulating synaptic plasticity in the entorhinal cortex.

The contribution of the calmodulin-dependent protein phosphatase calcineurin to LTD was tested by incubating slices in cyclosporin A or by including FK506 in the recording electrode solution. Cyclosporin A appeared to cause a partial block of LTD, and responses were reduced to 82.4% of baseline as compared to 58.6% in untreated cells (compare Figures 1(c) and 3(a)), but the sizes of these LTD effects were not statistically different. We obtained a more conclusive result with FK506, however, and LTD was completely blocked by including FK506 in the recording electrode solution. Including FK506 in the bathing medium has been used to block calcineurin-dependent depression effects in entorhinal cortex [28], and in excitatory [47] and inhibitory [48] synapses of the CA1 region. Here, we have loaded FK506 into the recording electrode solution to avoid possible presynaptic effects of the drug and to ensure that FK506 could act on calcineurin [39, 40, 49, 50]. The block of LTD by FK506 indicates that LTD is dependent on calcineurin, and this suggests that cyclosporin A resulted in only a partial block of calcineurin activity.

Calcineurin is thought to mediate expression of LTD in part by dephosphorylating inhibitor 1 and thereby increasing the activity of PP1 [2, 4, 39]. The PP1/PP2a inhibitor okadaic acid blocks LTD in the CA1 region [38, 40], and we have shown here that the induction of LTD in the entorhinal cortex was blocked by including okadaic acid in the recording electrode solution. This is the first report of LTD in the entorhinal cortex dependent on PP1/PP2a. Protein phosphatases can regulate synaptic function through a variety of mechanisms [51] that include dephosphorylation of the ser-845 residue on the AMPA GluR1 subunit, and LTD in the entorhinal cortex may be expressed partly through this mechanism. In addition, the work of Deng and Lei [28] has found entorhinal LTD to be associated with a reduction in the number of postsynaptic AMPA receptors, with no change in AMPA receptor conductance, and has shown that this effect is dependent on proteosomes that degrade AMPA receptors internalized through ubiquitinization. As in the hippocampus, therefore, entorhinal LTD can be expressed through mechanisms involving trafficking of AMPA receptors [52].

Long-term depression was induced here using strong repetitive paired-pulse stimulation which we have used previously to induce LTD in the entorhinal cortex in vivo and in slices ([17, 18], see also [33, 34]). LTD was induced following 15 minutes, but not 7.5 minutes of paired-pulse stimulation; this is consistent with a requirement for prolonged activation of calcium-dependent signalling mechanisms, and is also consistent with the possibility that NMDA receptor-dependent metaplastic changes early in the train may promote LTD induced by stimuli that occurred later in the 15-minute duration trains [53]. We previously found 1 Hz stimulation to be ineffective in vivo and in slices from Long-Evans rats [17, 18], but deep layer inputs to stellate neurons in slices from 2 to 3 week-old Sprague-Dawley rats express NMDA receptor-dependent LTD following 15 minutes of 1 Hz stimulation, or following low-frequency stimulation paired with postsynaptic depolarization [28]. Thus, there may be developmental, strain-related, or pathway-specific factors that affect the ability of 1 Hz stimulation to activate these signalling mechanisms.

The entorhinal cortex is embedded within the temporal lobe through an extensive array of anatomical connections [7] and has been linked behaviorally to a variety of sensory and cognitive functions (e.g., [9, 10]). Lasting synaptic plasticity in the entorhinal cortex is therefore likely to serve a variety of functions depending on the synaptic pathways involved. Synaptic depression effects are generally thought to complement synaptic potentiation during the formation of memory [45, 54–56], and it is possible that depression effects contribute to short and/or long-term memory processing. However, the laminar architecture of the entorhinal cortex, with superficial layers mediating much of the cortical input to the hippocampal formation, suggests that long-term depression of synaptic transmission in layer II may lead to long-term reductions in the salience of particular elements or patterns of cortical input and may thus lead to lasting changes in the multimodal inputs processed by the hippocampal formation. Similarly, the general resistance of the entorhinal cortex to induction of LTD could serve to maintain relatively stable information processing and integration of multimodal sensory inputs within the medial entorhinal cortex.

## References

[1] Malenka RC, Nicoll RA (1999). Long-term potentiation—a decade of progress?. *Science*.

[2] Malenka RC, Bear MF (2004). LTP and LTD: an embarrassment of riches. *Neuron*.

[3] Bear MF, Abraham WC (1996). Long-term depression in hippocampus. *Annual Review of Neuroscience*.

[4] Kemp N, Bashir ZI (2001). Long-term depression: a cascade of induction and expression mechanisms. *Progress in Neurobiology*.

[5] Massey PV, Bashir ZI (2007). Long-term depression: multiple forms and implications for brain function. *Trends in Neurosciences*.

[6] Burwell RD, Amaral DG (1998). Cortical afferents of the perirhinal, postrhinal, and entorhinal cortices of the rat. *Journal of Comparative Neurology*.

[7] Kerr KM, Agster KL, Furtak SC, Burwell RD (2007). Functional neuroanatomy of the parahippocampal region: the lateral and medial entorhinal areas. *Hippocampus*.

[8] de Curtis M, Paré D (2004). The rhinal cortices: a wall of inhibition between the neocortex and the hippocampus. *Progress in Neurobiology*.

[9] Barry C, Hayman R, Burgess N, Jeffery KJ (2007). Experience-dependent rescaling of entorhinal grids. *Nature Neuroscience*.

[10] Lipton PA, White JA, Eichenbaum H (2007). Disambiguation of overlapping experiences by neurons in the medial entorhinal cortex. *The Journal of Neuroscience*.

[11] Caruana DA, Reed SJ, Sliz DJ, Chapman CA (2007). Inhibiting dopamine reuptake blocks the induction of long-term potentiation and depression in the lateral entorhinal cortex of awake rats. *Neuroscience Letters*.

[12] Racine RJ, Milgram NW, Hafner S (1983). Long-term potentiation phenomena in the rat limbic forebrain. *Brain Research*.

[13] Chapman CA, Racine RJ (1997). Converging inputs to the entorhinal cortex from the piriform cortex and medial septum: facilitation and current source density analysis. *Journal of Neurophysiology*.

[14] Chapman CA, Racine RJ (1997). Piriform cortex efferents to the entorhinal cortex in vivo: kindling-induced potentiation and the enhancement of long-term potentiation by low-frequency piriform cortex or medial septal stimulation. *Hippocampus*.

[15] Alonso A, de Curtis M, Llinás R (1990). Postsynaptic Hebbian and non-Hebbian long-term potentiation of synaptic efficacy in the entorhinal cortex in slices and in the isolated adult guinea pig brain. *Proceedings of the National Academy of Sciences of the United States of America*.

[16] Yun SH, Mook-Jung I, Jung MW (2002). Variation in effective stimulus patterns for induction of long-term potentiation across different layers of rat entorhinal cortex. *The Journal of Neuroscience*.

[17] Bouras R, Chapman CA (2003). Long-term synaptic depression in the adult entorhinal cortex in vivo. *Hippocampus*.

[18] Kourrich S, Chapman CA (2003). NMDA receptor-dependent long-term synaptic depression in the entorhinal cortex in vitro. *Journal of Neurophysiology*.

[19] Dudek SM, Bear MF (1992). Homosynaptic long-term depression in area CA1 of hippocampus and effects of *N*-methyl-D-aspartate receptor blockade. *Proceedings of the National Academy of Sciences of the United States of America*.

[20] Mulkey RM, Malenka RC (1992). Mechanisms underlying induction of homosynaptic long-term depression in area CA1 of the hippocampus. *Neuron*.

[21] Dudek SM, Bear MF (1993). Bidirectional long-term modification of synaptic effectiveness in the adult and immature hippocampus. *The Journal of Neuroscience*.

[22] Wagner JJ, Alger BE (1995). GABAergic and developmental influences on homosynaptic LTD and depotentiation in rat hippocampus. *The Journal of Neuroscience*.

[23] Kemp N, McQueen J, Faulkes S, Bashir ZI (2000). Different forms of LTD in the CA1 region of the hippocampus: role of age and stimulus protocol. *European Journal of Neuroscience*.

[24] Errington ML, Bliss TV, Richter-Levin G, Yenk K, Doyere V, Laroche S (1995). Stimulation at 1–5 Hz does not produce long-term depression or depotentiation in the hippocampus of the adult rat in vivo. *Journal of Neurophysiology*.

[25] Doyle CA, Cullen WK, Rowan MJ, Anwyl R (1997). Low-frequency stimulation induces homosynaptic depotentiation but not long-term depression of synaptic transmission in the adult anaesthetized and awake rat hippocampus in vivo. *Neuroscience*.

[26] Staubli U, Scafidi J (1997). Studies on long-term depression in area CA1 of the anesthetized and freely moving rat. *The Journal of Neuroscience*.

[27] Heynen AJ, Abraham WC, Bear MF (1996). Bidirectional modification of CA1 synapses in the adult hippocampus in vivo. *Nature*.

[28] Deng P-Y, Lei S (2007). Long-term depression in identified stellate neurons of juvenile rat entorhinal cortex. *Journal of Neurophysiology*.

[29] Cheong MY, Yun SH, Mook-Jung I, Kang Y, Jung MW (2002). Induction of homosynaptic long-term depression in entorhinal cortex. *Brain Research*.

[30] Thiels E, Barrionuevo G, Berger TW (1994). Excitatory stimulation during postsynaptic inhibition induces long-term depression in hippocampus in vivo. *Journal of Neurophysiology*.

[31] Doyère V, Errington ML, Laroche S, Bliss TV (1996). Low-frequency trains of paired stimuli induce long-term depression in area CA1 but not in dentate gyrus of the intact rat. *Hippocampus*.

[32] Thiels E, Xie X, Yeckel MF, Barrionuevo G, Berger TW (1996). NMDA receptor-dependent LTD in different subfields of hippocampus in vivo and in vitro. *Hippocampus*.

[33] Solger J, Wozny C, Manahan-Vaughan D, Behr J (2004). Distinct mechanisms of bidirectional activity-dependent synaptic plasticity in superficial and deep layers of rat entorhinal cortex. *European Journal of Neuroscience*.

[34] Solger J, Heinemann U, Behr J (2005). Electrical and chemical long-term depression do not attenuate low-Mg^2+^-induced epileptiform activity in the entorhinal cortex. *Epilepsia*.

[35] Woodhall GL, Bailey SJ, Thompson SE, Evans DIP, Jones RSG (2005). Fundamental differences in spontaneous synaptic inhibition between deep and superficial layers of the rat entorhinal cortex. *Hippocampus*.

[36] Watabe AM, Carlisle HJ, O'Dell TJ (2002). Postsynaptic induction and presynaptic expression of group 1 mGluR-dependent LTD in the hippocampal CA1 region. *Journal of Neurophysiology*.

[37] Kemp N, Bashir ZI (1999). Induction of LTD in the adult hippocampus by the synaptic activation of AMPA/kainate and metabotropic glutamate receptors. *Neuropharmacology*.

[38] Mulkey RM, Herron CE, Malenka RC (1993). An essential role for protein phosphatases in hippocampal long-term depression. *Science*.

[39] Mulkey RM, Endo S, Shenolikar S, Malenka RC (1994). Involvement of a calcineurin/inhibitor-1 phosphatase cascade in hippocampal long-term depression. *Nature*.

[40] Morishita W, Marie H, Malenka RC (2005). Distinct triggering and expression mechanisms underlie LTD of AMPA and NMDA synaptic responses. *Nature Neuroscience*.

[41] Yang S-N (2000). Ceramide-induced sustained depression of synaptic currents mediated by ionotropic glutamate receptors in the hippocampus: an essential role of postsynaptic protein phosphatases. *Neuroscience*.

[42] Alonso A, Klink R (1993). Differential electroresponsiveness of stellate and pyramidal-like cells of medial entorhinal cortex layer II. *Journal of Neurophysiology*.

[43] van der Linden S (1998). Comparison of the electrophysiology and morphology of layers III and II neurons of the rat medial entorhinal cortex in vitro. *European Journal of Neuroscience*.

[44] Zhou Y-D, Acker CD, Netoff TI, Sen K, White JA (2005). Increasing Ca^2+^ transients by broadening postsynaptic action potentials enhances timing-dependent synaptic depression. *Proceedings of the National Academy of Sciences of the United States of America*.

[45] Christie BR, Kerr DS, Abraham WC (1994). Flip side of synaptic plasticity: long-term depression mechanisms in the hippocampus. *Hippocampus*.

[46] Haas JS, Nowotny T, Abarbanel HDI (2006). Spike-timing-dependent plasticity of inhibitory synapses in the entorhinal cortex. *Journal of Neurophysiology*.

[47] Kang-Park M-H, Sarda MA, Jones KH (2003). Protein phosphatases mediate depotentiation induced by high-intensity theta-burst stimulation. *Journal of Neurophysiology*.

[48] Lu YM, Mansuy IM, Kandel ER, Roder J (2000). Calcineurin-mediated LTD of GABAergic inhibition underlies the increased excitability of CA1 neurons associated with LTP. *Neuron*.

[49] Li S-T, Kato K, Tomizawa K (2002). Calcineurin plays different roles in group II metabotropic glutamate receptor- and NMDA receptor-dependent long-term depression. *The Journal of Neuroscience*.

[50] Yasuda H, Higashi H, Kudo Y (2003). Imaging of calcineurin activated by long-term depression-inducing synaptic inputs in living neurons of rat visual cortex. *European Journal of Neuroscience*.

[51] Mansuy IM, Shenolikar S (2006). Protein serine/threonine phosphatases in neuronal plasticity and disorders of learning and memory. *Trends in Neurosciences*.

[52] Bredt DS, Nicoll RA (2003). AMPA receptor trafficking at excitatory synapses. *Neuron*.

[53] Mockett B, Coussens C, Abraham WC (2002). NMDA receptor-mediated metaplasticity during the induction of long-term depression by low-frequency stimulation. *European Journal of Neuroscience*.

[54] Bear MF (1996). A synaptic basis for memory storage in the cerebral cortex. *Proceedings of the National Academy of Sciences of the United States of America*.

[55] Abbott LF, Nelson SB (2000). Synaptic plasticity: taming the beast. *Nature Neuroscience*.

[56] Braunewell K-H, Manahan-Vaughan D (2001). Long-term depression: a cellular basis for learning?. *Reviews in the Neurosciences*.

